# Prioritization, clustering and functional annotation of MicroRNAs using latent semantic indexing of MEDLINE abstracts

**DOI:** 10.1186/s12859-016-1223-2

**Published:** 2016-10-06

**Authors:** Sujoy Roy, Brandon C. Curry, Behrouz Madahian, Ramin Homayouni

**Affiliations:** 1 Bioinformatics Program, University of Memphis, Memphis, 38152 USA; 2 Center for Translational Informatics, University of Memphis, Memphis, 38152 USA; 3 Department of Mathematical Sciences, University of Memphis, Memphis, 38152 USA; 4 Department of Biology, University of Memphis, Memphis, 38152 USA

**Keywords:** MicroRNAs, Text mining, Latent semantic indexing, Singular value decomposition

## Abstract

**Background:**

The amount of scientific information about MicroRNAs (miRNAs) is growing exponentially, making it difficult for researchers to interpret experimental results. In this study, we present an automated text mining approach using Latent Semantic Indexing (LSI) for prioritization, clustering and functional annotation of miRNAs.

**Results:**

For approximately 900 human miRNAs indexed in miRBase, text documents were created by concatenating titles and abstracts of MEDLINE citations which refer to the miRNAs. The documents were parsed and a weighted term-by-miRNA frequency matrix was created, which was subsequently factorized via singular value decomposition to extract pair-wise cosine values between the term (keyword) and miRNA vectors in reduced rank semantic space. LSI enables derivation of both explicit and implicit associations between entities based on word usage patterns. Using miR2Disease as a gold standard, we found that LSI identified keyword-to-miRNA relationships with high accuracy. In addition, we demonstrate that pair-wise associations between miRNAs can be used to group them into categories which are functionally aligned. Finally, term ranking by querying the LSI space with a group of miRNAs enabled annotation of the clusters with functionally related terms.

**Conclusions:**

LSI modeling of MEDLINE abstracts provides a robust and automated method for miRNA related knowledge discovery. The latest collection of miRNA abstracts and LSI model can be accessed through the web tool miRNA Literature Network (miRLiN) at http://bioinfo.memphis.edu/mirlin.

**Electronic supplementary material:**

The online version of this article (doi:10.1186/s12859-016-1223-2) contains supplementary material, which is available to authorized users.

## Background

There is growing recognition that miRNAs regulate various diseases and biological processes [1–4] as evidenced by the rapidly growing body of literature related to miRNAs (Additional file 1: Figure S1). There are manually curated repositories such as miRBase [5] and miR2Disease [6] that catalog miRNAs in several organisms as well as summarize their associations with diseases and other biological processes. However, it is generally accepted that manual curation is unable to keep up with the rapidly growing genomic information [7]. For instance, miRBase has not been updated since 2014 and miR2Disease has not been updated since 2009. It is therefore imperative to devise automated methods that can keep pace with the functional information which is deposited in the biomedical literature with respect to miRNAs.

Information retrieval (IR) is a key component of text mining [8]. It consists of three types of models: set-theoretic (Boolean), probabilistic, and algebraic (vector space). Documents in each case are retrieved based on Boolean logic, probability of relevance to the query, and the degree of similarity to the query, respectively. The concept of literature-based discovery was introduced by Swanson [9] and has since been extended to many different areas of research. In the gene space, several approaches have focused on mining both explicit associations based on co-occurrence [10], as well as implicit associations based on higher order co-occurrence and indirect relationships [11].

Several IR approaches have focused on mining miRNA specific associations. miRCancer [12], miRSel [13] and miRTex [14] use co-occurrence and sentence level natural language processing to automatically extract direct relationships from text between miRNAs and genes or diseases. While useful, these tools may miss miRNA interactions where direct relationships were not explicitly stated. In such cases, automated extraction of semantic relationships would be useful to associate genes and miRNAs based on shared biological processes. Also, explicit relationships such as those based on co-occurrence count between miRNAs and genes may be harder to prioritize if they have the exact same score. In contrast, semantic associations that take into account other relationships could be useful for prioritization of miRNA and gene associations [15].

Aside from exploring miRNA to gene associations, semantic analysis could be useful for other research scenarios. For example, investigators may want to prioritize candidate miRNAs for specific diseases or phenotypes. Alternatively, investigators may want to understand the functional pathways shared between different miRNAs. To address these needs, we developed and evaluated an LSI based text mining approach. Previously, we applied LSI to extract functional relationships amongst genes [16] as well as relationships between genes and transcription factors [15] from MEDLINE abstracts. LSI uses Singular Value Decomposition (SVD) [17, 18], which is a dimensionality reduction technique that decomposes the original term-by-document weighted frequency matrix into a new set of factor matrices that can be used to represent both terms and documents in lower-dimensional subspace. Previously, we demonstrated that LSI can extract both explicit (direct) and implicit (indirect) semantic relationships amongst genes. In addition, LSI allows genes to be prioritized based on keyword queries as well as gene-abstract queries with better accuracy than term co-occurrence methods [16]. Here, we applied this approach to miRNAs and demonstrate its utility to prioritize, cluster and functionally annotate miRNAs. The accompanying web based tool, miRNA Literature Network (miRLiN), available at http://bioinfo.memphis.edu/mirlin, provides an automated framework for interactively extracting and discovering functional information on human miRNAs based on up to date biomedical literature.

## Methods

### miRNA document collection

For 1881 human miRNAs indexed in the miRBase repository, 3 different abstract collections were built. Firstly, a curated collection limited to manually assigned abstracts was constructed. A total of 8110 unique abstracts (citations) cross referenced in the linkouts from miRBase as well as Entrez Gene [19] were collected. These citations (identified by unique PubMed identifiers or PMIDs) have been assigned either by professional staff at the National Library of Medicine, or by the scientific research community via Gene Reference into Function (GeneRIF) portal, or by curators of miRBase. Since these abstracts are manually curated, they are expected to have a very high precision for tagging correct citations to miRNAs but at the same time the number of citations referenced for each miRNA is a small proportion of the total number of relevant citations in MEDLINE for that miRNA, resulting in low recall.

In order to increase the information content for the miRNAs, a retrieved collection was built by querying the PubMed repository. A single miRNA can be referenced in the literature in several spelling variants e.g., *mir19a*, *mir-19a*, *microRNA19a*, *microRNA-19a* etc. For each miRNA, all such tentative synonyms with and without hyphens were constructed, and a PubMed query with the form ‘ synonym #1 OR synonym #2 OR...OR synonym #n’ was submitted using the NCBI efetch utility for retrieving relevant citations that have at least one synonym present in either title or abstract. Further restrictions were added to the query to limit the search to abstracts relevant to humans and miRNAs. A total of 19191 unique citations were retrieved.

The two collections were merged to get 19527 unique citations. We further filtered the nonspecific citations by removing PMIDs that referred to 7 or more miRNAs. Typically, these citations described sequencing experiments which mentioned a large number of miRNAs without substantive biological or mechanistic information. This threshold of 7 was derived as the smallest right outlier in the distribution of numbers of miRNAs linked to each unique citation. The outlier calculation was based on the IQR (interquartile range). The IQR is *Q*3 (75^th^ percentile) – *Q*1 (25^th^ percentile). The designated outliers were >*Q*3+1.5∗*I* *Q* *R*. Post filtering, 17076 unique citations and 878 active miRNAs (the ones referenced by at least one citation) remained in the collection, which comprised of less than half of the original number of 1881 miRNAs. Thus a large number of miRNAs were excluded from our collection because they lacked a specific citation. The number of citations assigned to the active miRNAs ranged from 1 (28 % of the collection) to 1451. The average and median number of citations in the collection were 38 and 4, respectively. For each of 878 active miRNAs, a miRNA document was created by concatenating the titles and abstracts of all citations referenced by the miRNA.

### Construction of the LSI model

**Fig. 1:**
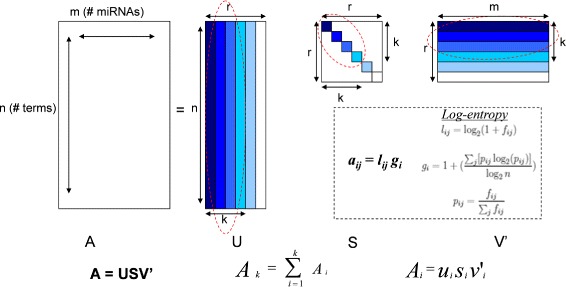
Overview of Latent Semantic Indexing. In a vector-space model, the semantic structure of a document is represented as a vector (essentially, a bag of words) in word space, and the degree of similarity between documents is calculated by the cosine of the angle between document vectors. The vectors consist of weighted terms, which are a function of the frequency of the terms in and across all documents in the collection. A variant of the vector space model, called Latent Semantic Indexing, improves retrieval by applying singular value decomposition (SVD) to create a subspace in which text documents are represented as vectors. The components in the subspace may be regarded as a concept derived from the word usage patterns in the document. Hence, the relevant documents are retrieved based on the degree of conceptual similarity between the documents

The outline of the LSI approach used in this study is depicted in Fig. 1. Sixty eight thousand five hundred ninety-six terms (keywords) were parsed from the collection of 878 miRNA documents using Text to Matrix Generator software [20]. All punctuation (excluding hyphens and underscores) and capitalization were ignored and, in addition, articles and other common, non-distinguishing words were discarded using the stop list from Cornell’s SMART project repository [21]. A term-by-miRNA matrix was created where the entries of the matrix were log-entropy weighted frequencies of terms across the miRNA document collection. Term weighting schemes are typically employed in order to normalize the matrix and discount the effect of common terms while at the same time increasing the importance of terms that are better delineators between miRNA documents. Each matrix entry *a*_*ij*_ is transformed into a product of a local component (*l*_*ij*_) and global component (*g*_*i*_):

1 $$ l_{ij} = \log_{2}(1 + f_{ij})  $$

2 $$ g_{i} = 1 + \frac{\sum_{j}p_{ij}\log_{2}p_{ij}}{\log_{2}n}  $$

3 $$ p_{ij} = \frac{f_{ij}}{\sum_{j}f_{ij}}  $$

where *f*_*ij*_ is the frequency of the *i* ^th^ term in the *j* ^th^ miRNA-document, *p*_*ij*_ is the probability of the *i* ^th^ term occurring in the *j* ^th^ miRNA-document and *n* is the number of miRNA documents in the collection. The log-entropy weighting scheme is based on information-theoretic concepts and takes into account the distribution of terms over miRNA documents and has been found to be more useful in extracting implied relationships [22].

Singular value decomposition (SVD) [17, 18] was applied to the term-by-miRNA log-entropy weighted frequency matrix. A data matrix *A* with *n* rows (terms) and *m* columns (miRNAs), where *n*>>*m*, can be construed as *n* term row vectors in *m*-dimensional miRNA space and *m* miRNA-document column vectors in *n*-dimensional term space. SVD transforms the two sets of vectors into a new *r*-dimensional orthogonal space in which the maximum variation is expressed along the first dimension axis, as much variation independent of that is expressed along an axis orthogonal to the first, and so on. The new set of axes may reveal the true dimensionality of the data if the dataset is not inherently *m*-dimensional. The SVD is formulated as:

4 $$ A = USV'  $$

where ^′^ indicates transpose of the matrix obtained by permuting the modes, i.e., transforming rows into columns and vice versa, *U* is *n*×*r*, *S* is *r*×*r*, and *V* is *m*×*r* (*V* ^′^ is *r*×*m*). Both *U* and *V* are orthogonal, i.e., *U* *U* ^′^=*I* and *V* *V* ^′^=*I* where *I* is the identity matrix. *S* is a diagonal matrix with non-negative and non-increasing entries *σ*_1_,*σ*_2_,...,*σ*_*r*_ which are known as singular values. *r* is the rank of the matrix, which is the number of linearly independent rows or columns of *A*. It is however, known from observation, for most practical datasets, *r*=*m*. The third matrix *V* is written as a transpose so that the rows of both matrices *U* and *V* correspond to terms and miRNAs, respectively.

The rows of *A* can be interpreted as term coordinates in an *m*-dimensional space. The axes of this space can be interpreted as rows of *I* (identity matrix). The SVD transforms the term coordinates to rows of *U* and the axes to the rows of *S* *V* ^′^. The matrix *V* ^′^ acts as the rotation matrix for the original axes and the diagonal of matrix *S* contains the scaling factor for each axis. The *U* matrix can now be construed as a new transformed dataset whose rows still correspond to the original *n* terms but the miRNAs are transformed into *r* eigen miRNAs (factors) that are a linear combination of the original miRNAs.

SVD is symmetric in the sense that a decomposition on the rows (terms) can be transformed into a decomposition on the columns (miRNAs):

5 $$ A' = VSU'  $$

*A* ^′^ reverses the roles of terms and miRNAs. *V* plays the role originally played by *U* and *U* plays the role originally played by *V*. Since *S* is diagonal, *S*=*S* ^′^. The SVD transforms the miRNA coordinates to rows of *V* and the axes to the rows of *S* *U* ^′^. The matrix *U* ^′^ acts as the rotation matrix for the original axes and the diagonal of matrix *S* contains the scaling factor for each axis. The *V* matrix can now be construed as a new transformed dataset whose rows still correspond to the original *m* miRNAs but the terms are transformed into *r* eigen terms (factors) that are a linear combination of the original terms.

The new scaled and rotated axes and the coordinates tend to better fit the data than the original axes and coordinates. The singular values in *S* determine the relative importance of each axis. The first few axes capture the maximum variation in the data and the subsequent ones less so. Only the first *k* (where *k*<*r*) factors corresponding to *k* largest singular values may be used to represent the data. There are two potential benefits of performing this truncation. Firstly, for large datasets (with many attributes), this translates into savings in memory space as well as analysis time, as vectors in *k* dimensions can be compared in less time than vectors in *m* dimensions. Secondly, SVD reveals the true dimensionality present in the data, where the bulk of the information content in the original *m*-dimensional data may be captured in a lower dimensional manifold, after axis rotation and scaling.

An appropriate choice for *k* (number of most significant factors) can be made by assessing the contribution of each of the singular values as a measure of the amount of variation captured in each dimension, and then calculating the entropy of the contributions that might be indicative of what percentage of the total number of factors may be needed [23]. The contribution *C*_*i*_ of each of *r* singular values *σ*s can be calculated as:

6 $$ C_{i} = \frac{{\sigma_{i}^{2}}}{\sum_{i=1}^{r}{\sigma_{i}^{2}}}  $$

and the entropy of the *r* contributions calculated as:

7 $$ E = \frac{-1}{\log r} \sum_{k=1}^{r} C_{k}\log C_{k}  $$

Entropy measures the amount of disorder in the set of variations captured in the *r* dimensions. The magnitude of the entropy may vary from 0 (all variation is captured in the firrst dimension) to 1 (all dimensions are equally important). *k* is calculated as *E*×*r*. For the term-by-miRNA matrix, *k* was computed to be 560.

The association between any pair of entities (term-term, term-miRNA, miRNA-miRNA) can be calculated as the cosine of the angle between the respective *k*-dimensional vectors. The association scores can theoretically fall between −1 and 1, but in practice were observed to occur between −0.2472 and 1. A higher association score between a pair of entities indicates a stronger relationship in literature.

### Evaluation

#### Information Gain calculation

Information gain (in context of citations) for each miRNA was calculated as

8 $$ {{\begin{aligned} {}\frac{\# of\ \!citations\ retrieved\ from\ PubMed \!-\#\ \!of\ \!citations\ in\ miRBase\ and\ Entrez\ \!Gene}{\# of\ citations\ in\ miRBase\ and\ Entrez\ Gene} \end{aligned}}}  $$

#### Gold standards

miR2Disease was used for evaluating LSI performance. It is a comprehensive database containing descriptions of more than 100 diseases and their associated miRNAs.

#### AUC calculation

The term-to-miRNA and miRNA-to-term prioritizations were evaluated against gold standards by generating Receiver Operating Characteristics (ROC) curves which display recall and false positive rates at each rank. The area under the curve (AUC) can be used as a measure of ranking quality [24, 25]. The AUC will have the value of 1 for perfect ranking (all relevant entities at the top), 0.5 for randomly generated ranking, and 0 for the worst possible ranking (all relevant entities at the bottom).

#### Cohesion calculation

The cohesion for a set of miRNAs was calculated as described in [11, 26]. Given a set of *n* miRNAs for a disease, *n* AUCs were calculated. Each miRNA was treated as a query and the rest of the *n*−1 miRNAs were treated as gold standard. The set of all miRNAs (for all diseases) in miR2Disease were prioritized against the query miRNA using the cosine between the miRNA vectors as the similarity measure, and an AUC was calculated. The median AUC out of *n* AUCs was treated as the cohesion. If a set of miRNAs for a disease are closely related, then the miRNAs in the set would ideally have high cosine association with each other compared to remaining miRNAs that are not in the set, signifying a highly cohesive set.

## Results

### miRNA Literature Landscape

**Fig. 2:**
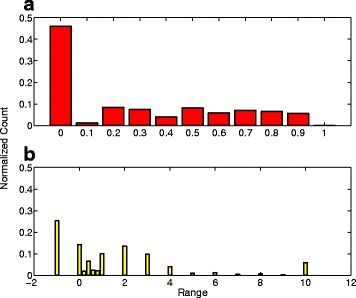
Distribution of recall (**a**) and information gain (**b**) metrics for curated and retrieved citations for miRNAs. Curated citations were collected from miRBase and Entrez Gene. The retrieved citations were obtained by querying PubMed using a compound search term, which included the miRNA symbol and its aliases. Recall for a miRNA was computed as the fraction of curated citations present amongst the retrieved citations for that miRNA. Information gain for a miRNA was calculated as the ratio between the number of additional citations retrieved, and the number of curated citations for that miRNA

The annual number of publications related to miRNAs is growing exponentially. This trend is observed in curated databases such as miRBase and Entrez Gene, as well as in PubMed using “miRNA” keyword search (Additional file 1: Figure S1). Overall, 2.37 times more citations were retrieved from the PubMed search than the number designated in curated databases. To collect more abstracts for the growing number of miRNAs, we designed an automated search strategy as described in “Methods”. Out of 1881 miRNAs found in miRBase, while all had at least one manually designated citation in either miRBase or Entrez Gene, only 974 had at least one citation retrieved from PubMed. Our PubMed search did not identify abstracts for nearly 50 % of the miRNAs in the curated databases. For the aforementioned 974 miRNAs with at least one retrieved citation, the recall values for more than 50 % of miRNAs were between 0.1 and 0.9 when using the curated citations as gold standard (Fig. 2 a). It is however important to note that our PubMed search strategy retrieved 95.8 % of all abstracts in curated databases (Additional file 1: Figure S2). This result suggests that there may be discrepancies in the curated databases for assignment of citations to miRNAs. On the other hand, it is possible that our search strategy misses important aliases for some miRNAs, thus affecting the recall performance. Next, we calculated the information gain, as described in Methods, for each of the 974 miRNAs. 589 miRNAs showed positive information gain and 304 miRNAs showed a negative information gain (Fig. 2 b). Only 55 miRNAs showed an information gain greater than 10. Based on these results, we concluded that merging citations from miRBase and Entrez Gene with PubMed retrieved citations would allow for the best coverage and information gain for building the LSI model.

**Fig. 3:**
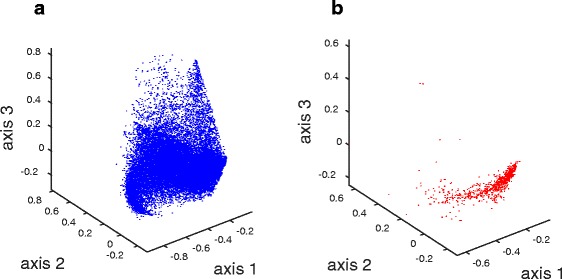
Distribution of term vectors (**a**) and miRNA vectors (**b**) across the first three LSI dimensions. Each point on the plots represents a single term or miRNA. For each vector, the magnitude of each axis component was scaled by the corresponding singular value and the scaled vector was then normalized to unit length

Once the abstract collection was updated and filtered for all miRNAs, an LSI model was built using a total of 17076 citations for the remaining 878 human miRNAs, as described in the “Methods”. Figure 3 shows the first three dimensions of the normalized scaled term vectors (A) and miRNA vectors (B) in LSI space. Both term and miRNA vectors are comparable with each other as they share the same coordinate space. We found that while term vectors span a broad area, the miRNA vectors are more concentrated. The limited distribution of the miRNA vectors suggests that the documents share many terms and that miRNAs are functionally quite similar. Additional file 1: Figure S3 shows the distribution of normalized singular values. The first factor captured a little more 3 % of the variance (information content) of the term-by-miRNA matrix. For this study, we used the top 560 (64 %) factors out of 878 factors, which comprised 93 % of the total information content.

### Evaluation of the LSI model

LSI is a robust approach to extract both explicit and implicit relationships between terms and miRNAs directly from the biomedical literature. In this study, the performance of the LSI model was evaluated based on three different use-case scenarios as described below.

#### miRNA ranking by term query

**Fig. 4:**
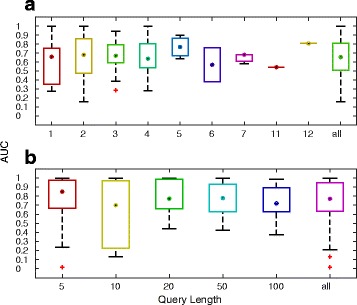
Distribution of area under the Receiver Operating Characteristic Curve (AUC) for term-to-miRNA (**a**) and miRNA-to-term (**b**) rankings. For term-to-miRNA rankings, the terms constituting a given disease name (obtained from the miR2Disease knowledge base) were used as the query, the query length refers to the number of terms, and the miRNAs associated with the disease were utilized as the gold standard. For miRNA-to-term rankings, the miRNAs associated with a given disease were used as the query, the query length refers to the number of miRNAs, and the terms constituting the disease name were utilized as the gold standard. For both types of rankings, the AUC values are shown stratified across various query lengths

A typical use-case involves ranking relevant miRNAs based on their association with keyword queries. A query may consist of a single word (term) such as “cancer” or a combination of words such as “head and neck squamous cell carcinoma”. A binary query vector *q*_0_ of a length equal to the total number of terms is created, with 1’s corresponding to the query terms and 0’s for the remaining terms in the dictionary. A term query *q* is constructed by projecting *q*_0_ onto the *U*_*k*_ matrix as *q*0′*U*_*k*_, which is the weighted sum of *k*-dimensional term vectors corresponding to the query terms in the *U*_*k*_ matrix. The miRNAs are prioritized by calculating the cosines of the term query with each of the scaled *k*-dimensional miRNA vectors in the *V* *S*_*k*_ matrix. To evaluate the LSI model, we used the miR2Disease knowledge base as the gold standard. Since miR2Disease was last updated in 2009, an LSI model specifically for this gold standard was generated using only publications dating to 2009 or earlier. For each disease, the full name (or descriptor) served as the query. Figure 4 shows the distribution of AUCs for different query term lengths. A full list of diseases and their respective AUCs are included in Additional file 2: Table S1A. The AUCs for 66 (56 %) of all disease queries were above 0.7. Generally, single word queries performed somewhat better than multiword queries. This result is expected as summing various term vectors could make the query ambiguous, and the high ranked miRNA vectors may actually be close to the composite query vector but only remotely related to any of the constituent terms of the composite query. In addition, disease categories which included more than 50 miRNAs generally resulted in lower AUCs. This may be due to the fact that some miRNAs may have multiple roles and molecular functions, thereby lowering their relative ranking against a single disease query. Lastly, these results may be in part due to discrepancies in the annotations by the curators of miR2Disease database.

To address the latter issue, we also tested the performance of the LSI model using a different gold standard (Gold Standard II) list of miRNAs for nine different diseases or physiologies. The gold standard II miRNAs were determined by manual examination of recent review papers on each topic (Table 1). The number of miRNAs in each disease category ranged from 8 to 43. The LSI model for evaluation was built using a collection of abstracts up to 2015. Importantly, we found that the average AUC for the nine disease queries was 0.89 (range = 0.80 to 0.94). These results were substantially higher than those achieved by using miR2Disease categories as gold standards, suggesting that miR2Disease database may have errors.

**Table 1:** Performance of the LSI model on disease or physiology term queries against expert determined gold standards culled from review papers

Name	# Terms in	# miRNAs in	AUC
	query	Gold Std.	
Cholesterol Homeostasis [33]	2	19	0.94496
Endothelium (endothelial) [34]	2	43	0.83294
Adipogenesis [35]	1	18	0.92189
Macrophage [36]	1	9	0.93975
HDL high density lipoprotein [37]	4	13	0.83344
Asthma [38]	1	9	0.87289
COPD Chronic obstructive pulmonary disease [38]	5	8	0.80491
Cystic Fibrosis [38]	2	10	0.93897
Idiopathic pulmonary fibrosis [38]	3	8	0.90993

#### Term ranking by miRNA query

Another use-case for researchers would be to functionally annotate groups of miRNAs. This is relevant to genomic experiments which generally yield many differentially expressed miRNAs. Here, the miRNAs are treated as the query and the relevant terms are rank ordered. miR2Disease was used to select groups of miRNAs that were assigned to specific diseases. To evaluate the performance of the LSI model, the top 300 ranked terms associated with the group of miRNAs were compared to the disease descriptors in miR2Disease database. A threshold of 300 terms was chosen because it would be impractical for users to consider the entire prioritized list of 68596 terms and also to reduce the computational burden. The list of diseases and their respective term AUCs are available in Additional file 2: Table S1B. The AUCs for 59 diseases could not be obtained as none of the constituent terms in the names of these diseases were found amongst the top 300 ranked terms. Among the queries which returned at least one disease term in the top 300 ranked terms, 27 (46 %) queries produced an AUC above 0.8. Surprisingly, the average AUC for the gold standard II list was 0.54 and none of the disease queries produced and AUC above 0.8 (Additional file 2: Table S2A). These results suggest that the top 300 terms extracted from the LSI model may be related to other topics (such as molecular functions etc.) than only diseases.

#### miRNA ranking by miRNA query

**Fig. 5:**
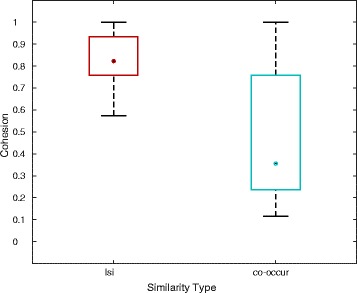
Distribution of cohesions for 122 diseases’ miRNA groups in miR2Disease. The cohesions for the LSI model were compared with those from the co-occurrence model. In the LSI model, the similarity between any two miRNAs was calculated as the cosine of the angle between their vectors in truncated LSI space. In the co-occurrence model, the similarity between any two miRNAs was designated as the number of shared citations

A third use-case is prioritization of miRNAs in response to a miRNA query. To evaluate the LSI model, we calculated the cohesion, as described in Methods, amongst the group of miRNAs assigned to specific diseases in miR2Disease (gold standard I) or by subject matter experts (gold standard II). The intent was to determine how well the LSI cosine similarity measure captures the real world clustering of related miRNAs. If a set of miRNAs are involved in a disease, then the miRNAs in the set should ideally have high cosine association with each other compared to remaining miRNAs that are not in the set. Figure 5 shows the distribution of cohesions for 122 miRNA disease groups having at least two miRNAs. The median cohesion for the LSI model was 0.83, compared to the median cohesion of 0.36 for a co-occurrence method, in which the similarity measure between miRNAs was designated as the number of shared abstracts. For 88 (72 %) diseases, the cohesions derived via the LSI model were significantly higher than chance when compared with the cohesions derived via the co-occurrence model (*p*≤0.05, ranksum test) (Additional file 2: Table S1C). In contrast, the median cohesion using LSI was only marginally better than that produced via the co-occurrence method using the gold standard II set, 0.577 and 0.576 respectively (Additional file 2: Table S2B). These results suggest that as the body of literature grows, miRNAs may be associated with many different pathways and functions beyond just specific diseases.

### Clustering and functional annotation of miRNAs

**Fig. 6:**
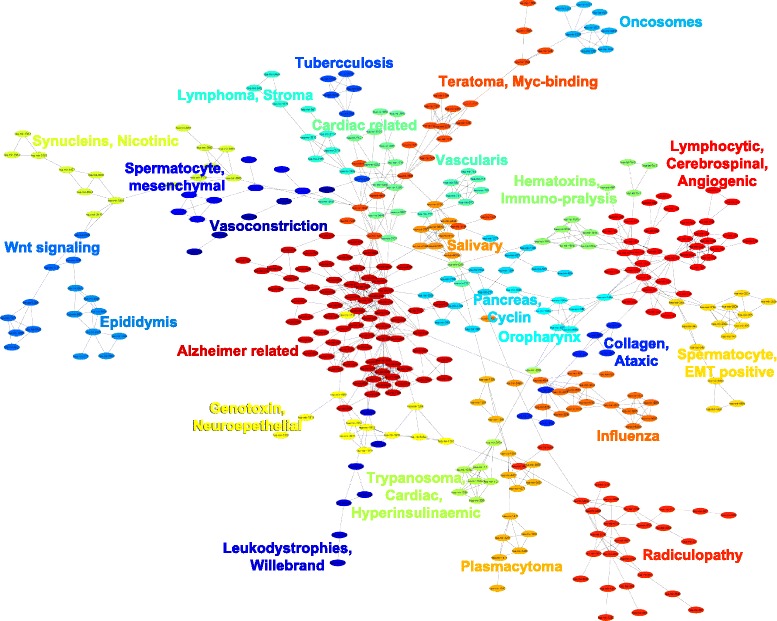
Clustering and functional annotation of miRNAs based on LSI derived associations. UKmeans clustering of the maximally connected component (∼350 miRNAs) of the miRNA graph, in which an edge is assigned if the cosine value is above 99^th^ percentile of all pair-wise cosine values. The functional annotations for each miRNA cluster were selected from amongst the top 300 ranked terms obtained via querying the truncated LSI space with the miRNA cluster

A major advantage of LSI is that the semantic relationships amongst all miRNA documents may be measured in lower dimensional (concept) space. Therefore, the cosine values between miRNAs may be used as a similarity score to cluster functionally related miRNAs. In addition, using the LSI model, miRNA clusters may be annotated using the top ranked terms as demonstrated above. Cosines were calculated for all miRNA pairs and a miRNA-miRNA cosine matrix was generated. The matrix was transformed into an adjacency (binary) matrix using a cosine threshold of 99 percentile of all pair-wise cosines (0.41715). The adjacency matrix was truncated to include only 365 miRNAs that were part of the largest connected component. The graph was clustered using UKmeans algorithm [27] with *k* = 25 to generate 25 mutually connected clusters (Fig. 6, Additional file 2: Table S3). For each cluster, the LSI model was queried using all of its miRNAs and then the top 300 terms were extracted for each cluster.

The terms were manually examined and used to label each cluster in Fig. 6. For instance, the largest cluster containing 73 miRNAs is associated with Alzheimer disease. This number is slightly different from the number (64 miRNAs) of Alzheimer related miRNAs in miR2Disease database. Interestingly, the largest miR2Disease group of miRNAs (152) was associated with hepatocellular carcinoma. It is important to note that the top nine miR2Disease categories, containing between 114 to 152 miRNAs, were all associated with some form of cancer. This suggests that there is a large bias in the miRNA databases as of 2009. By comparison, we found that the LSI-based clusters contained smaller number of miRNAs that were associated with more specific terms, which were functionally aligned. These results indicate that LSI based clustering allows for more robust functional clustering and more specific functional annotation beyond simply assigning miRNAs to diseases.

### miRLiN web tool

**Fig. 7:**
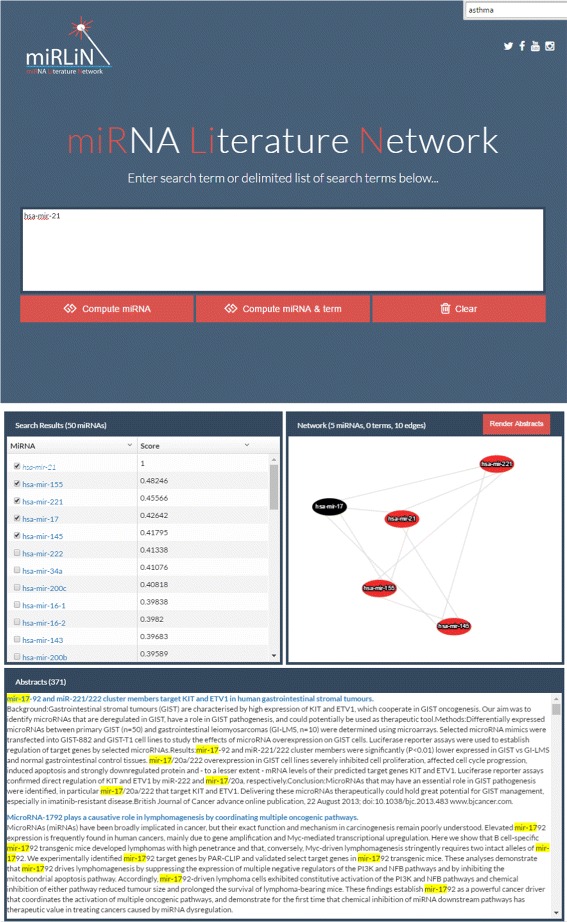
Screenshot of miRNA Literature Network (miRLiN) web tool. miRLiN enables users to prioritize miRNAs and terms according to queries. Specific miRNAs or terms can be selected (*upper left* panel) and displayed as a graph (*upper right* panel). Single or multiple nodes on the graph may be selected to view the abstracts associated with them in the lower panel

We developed a publicly available web-tool (http://bioinfo.memphis.edu/mirlin) to provide access to the LSI model, which contains the most recent and comprehensive collection of miRNA abstracts in MEDLINE (Fig. 7). The user can query the model with any combination of terms or miRNAs. When querying with terms, the tool ranks all miRNAs in the collection with respect to semantic associations to the query. Alternatively, a miRNA query may be used to compute associations with both miRNAs and terms. The output of the tool is a ranked list of miRNAs and terms based on the degree of association (cosine value) to the query. Selected miRNAs and terms can be visualized as a network graph, where the nodes represent the selected miRNAs and terms and the edges represent cosine values above 0.4. Multiple nodes can be selected from the graph display to retrieve their shared abstracts, if applicable. The abstracts are displayed with the selected terms and miRNAs highlighted for convenience.

For benchmarking, we compared the performance of our web tool with two existing web tools, miRCancer [12] and miRiaD [28]. While both tools are disease focused, miRLiN is more flexible and can accept any type of query. Also, both tools rely on databases with binary associations between miRNAs and diseases. In contrast, miRLiN ranks miRNAs based on the functional relevancy to the query and also enables a genome-wide network view of miRNAs with multiple associations to one another. Additional file 2: Tables S4A and S4B compare the results from the 3 web tools for ‘choriocarcinoma’ and ‘meningioma’ queries. For the ‘choriocarcinoma’ query, miRCancer listed 3 miRNAs (*hsa-mir-199b*, *hsa-mir-218*, *hsa-mir-34a*) while miRiaD listed 2 additional miRNAs (*hsa-mir-141*, *hsa-mir-126*). Importantly, miRLiN retrieved all 5 miRNAs within the top 15 ranked miRNAs (Additional file 2: Table S4A). We manually evaluated the 10 additional miRNAs retrieved by miRLiN. We found that miRNAs *hsa-mir-378a*, *hsa-mir-371b*, *hsa-mir-371a*, *hsa-let-7g*, *hsa-mir-373*, *hsa-mir-141* and *hsa-mir-15a* were co-mentioned with the query term in the same abstracts, but not in the same sentences. It appears that these miRNAs were found to be differentially expressed in choriocarcinoma cell lines. One miRNA *hsa-mir-145* was co-mentioned with the query term in the same sentence that suggests a direct link. Interestingly, *hsa-mir-585* association with choriocarcinoma appeared to be indirect via its association with *hsa-mir-218*. In addition, the abstract for *hsa-mir-141* in miRLiN was different from the other two web tools, suggesting that our abstract retrieval approach is slightly different than the other two methods. Lastly, *hsa-mir-624* did not appear to be related to choriocarcinoma or any other type of cancer, thus appears to be a false discovery.

For the ‘meningioma’ query, miRCancer retrieved 4 miRNAs (*hsa-mir-128*, *hsa-mir-200a*, *hsa-mir-224*, *hsa-mir-335*) and miRiaD retrieved 4 additional miRNAs (*hsa-mir-145*, *hsa-mir-190*, *hsa-mir-219*, and *hsa-mir-29*). Only two meningioma related miRNAs overlapped between miRiaD and miRCancer. In comparison, miRLiN retrieved all but one (*hsa-mir-145*, ranked 25^th^) amongst the top 12 ranked miRNAs (Additional file 2: Table S4B). Moreover, miRLiN identified two additional miRNAs (*hsa-mir-4417* and *hsa-mir-185*). Manual examination found that *hsa-mir-185* is in fact negatively associated with meningioma, where the citation explicitly negates its involvement in meningioma. This result reveals a shortcoming of our method, which does not take into account negations and other parts of speech that are considered in NLP based approaches. Lastly, manual examination did not find an association between *hsa-mir-4417* and meningioma, albeit it is associated with other types of cancer.

## Discussion

We have developed an LSI based approach to prioritize, cluster and functionally annotate miRNAs. LSI enables representation of miRNAs and terms as vectors in low dimensional space that can be compared against each other. LSI provides an advantage over co-occurrence based methods as semantic associations between entities take into account not only the entities being compared but also indirect associations amongst all other related entities in the collection. Several choices were made in the construction of the model that affects its performance. The rationale behind the choices and the potential ramifications of the alternatives are discussed below.

While building the miRNA document collection, citations that referenced more than 7 miRNAs were filtered out. Manual examination of citations revealed that certain high throughput screening papers were associated with many miRNAs but these papers did not describe any functional information about the specific miRNAs. For instance, many citations described sequencing experiments that identified several miRNAs. Inclusion of such citations in the model would create strong semantic associations between pairs of miRNAs that are otherwise remotely related. Better automated methods are needed to identify and filter such abstracts that do not describe any functional relationships.

Our results suggest that parsing of terms from miRNA documents still needs improvement. We found that many of the top 300 terms associated with groups of miRNAs were indeed too specific, relating to gene symbols or non-standard abbreviations used in the papers. For the current LSI model, only designated stopwords were removed prior to factorization. Automated methods may need to be investigated that can filter out additional non-useful terms. Stemming of the terms to their roots may also be useful in terms of reducing the dictionary size, although strategies for expanding the roots to the most relevant expansion will need to be devised once the terms are to be used for functional annotation. Currently, the selection of interesting functional annotation terms is still manual but could be automated by restricting to MeSH [29], GO [30] and KEGG [31]. However, this filtering strategy may result in loss of interesting terms such as gene or transcription factor names or phrases like ‘acaa-deletion’ that may indirectly link the miRNAs to a physiology or a biological function or a disease.

Several other methods may need to be investigated in the future to improve the performance of the LSI approach. For instance, different types of normalization methods for the term-by-miRNA matrix, in addition to the log-entropy method, may need to be investigated [22]. In the current study, an entropy based method was used to select *k* highest magnitude singular values. Other strategies have been discussed in the literature that may improve performance [32]. The web tool currently displays top 50 miRNAs and 300 terms in response to a query. Automated methods, such as one used for determining the singular value threshold, may also be useful in devising a prioritization threshold for cosines. Finally, adding collections for other model organisms such as mouse, rat etc. will make a more comprehensive text mining database for miRNAs.

## Conclusions

All together, we have demonstrated that an LSI based approach provides a robust and automated method to interrogate the large amount of literature that is accumulating with respect to miRNAs. The approach enables rapid prioritization of miRNAs in relation to keyword or miRNA queries. Furthermore, the LSI based approach allows for global clustering of all miRNAs based on functional information in the literature and provides a method for annotating groups of miRNAs with highly specific terms and concepts.

## Additional files

Additional file 1 Figures S1, S2, and S3. ’S10-S2.pdf’ contains supplementary figures 1, 2 and 3 in separate pages. (PDF 159 KB)

Additional file 2 Tables S1A, S1B, S1C, S2A, S2B, S3, S4A and S4B. Microsoft Excel 2013 workbook ’S11-S1.xlsx’ contains supplementary tables 1A, 1B, 1C, 2A, 2B, 3, 4A and 4B in separate tabs. (XLSX 32.5 KB)
