# Supplementary material for: Prioritization, clustering and functional annotation of MicroRNAs using latent semantic indexing of MEDLINE abstracts
Source: BMC Bioinformatics. 2016 Oct 6;17(Suppl 13):350. doi: 10.1186/s12859-016-1223-2 (PMC5073981; doi:10.1186/s12859-016-1223-2)
Supplement: Additional file 1 — Figures S1, S2, and S3. ’S10-S2.pdf’ contains supplementary figures 1, 2 and 3 in separate pages. (PDF 159 KB) [file 12859_2016_1223_MOESM1_ESM.pdf]

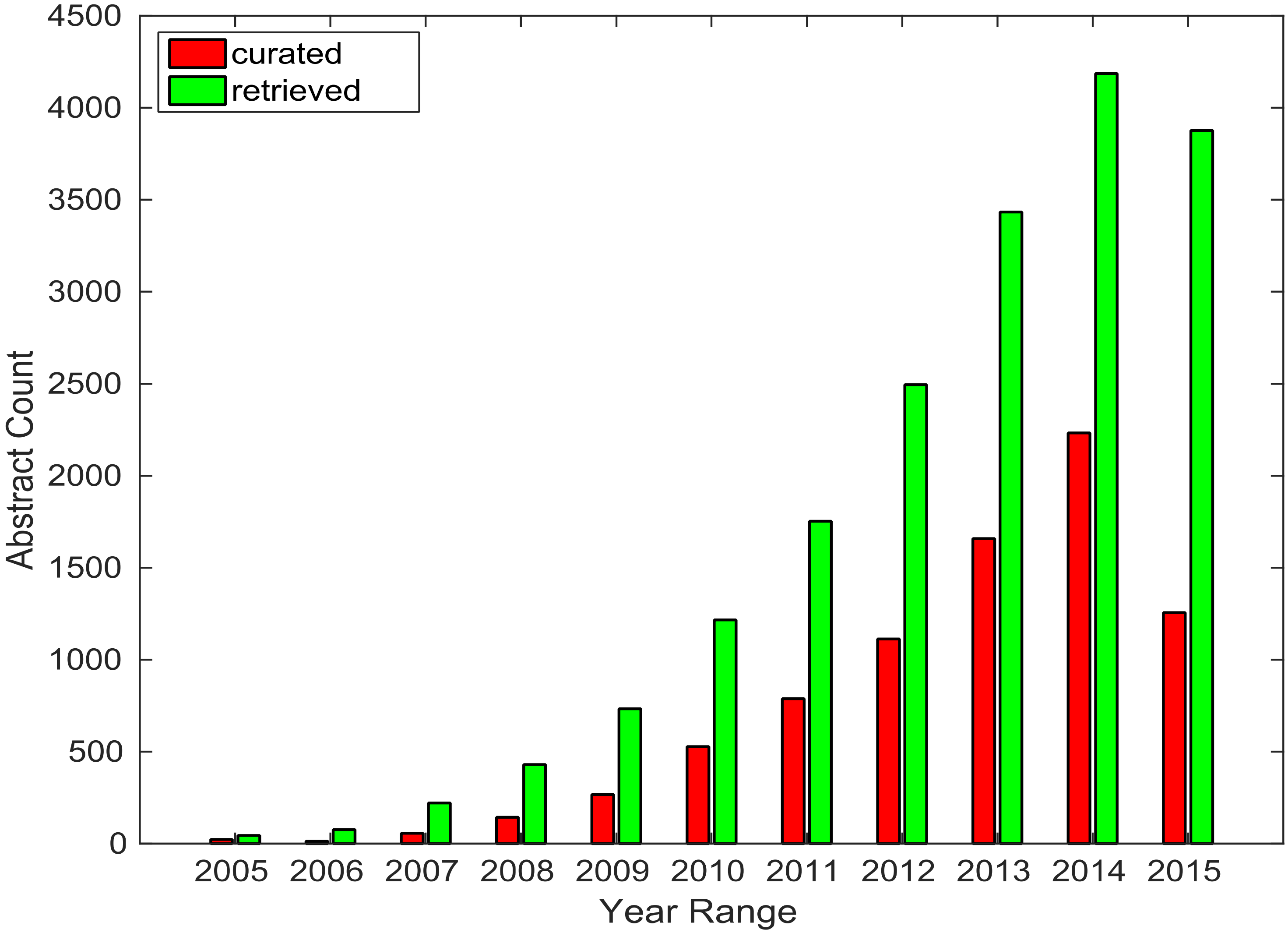

Fig. S1 Number of human miRNA abstracts by year, for curated and retrieved collections (prior to filtering).

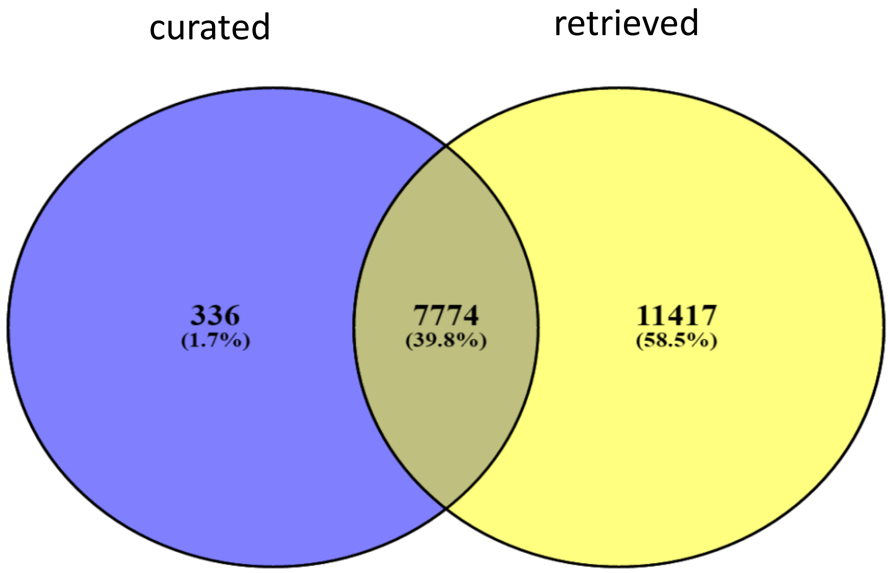

Fig. S2 Overlap between citations from curated and retrieved collections (prior to filtering).

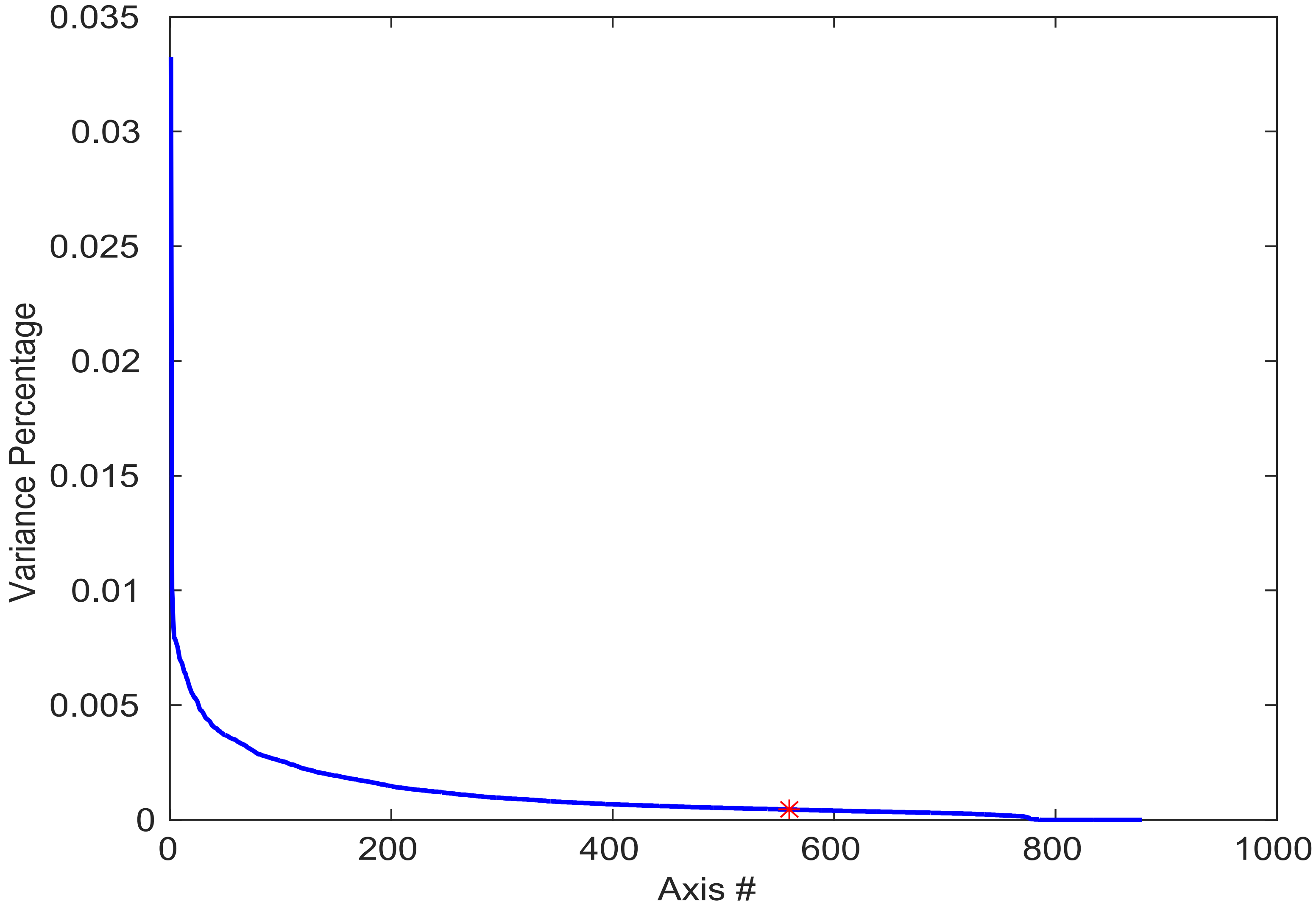

Fig. S3 Percentage of variance captured by each factor (axis).
